# Klotho gene delivery ameliorates renal hypertrophy and fibrosis in streptozotocin-induced diabetic rats by suppressing the Rho-associated coiled-coil kinase signaling pathway

**DOI:** 10.3892/mmr.2015.3367

**Published:** 2015-02-17

**Authors:** MINGHONG DENG, YUMEI LUO, YUNKUI LI, QIUCHEN YANG, XIAOQIN DENG, PING WU, HOUXUN MA

**Affiliations:** 1 Department of Geriatrics, The First Affiliated Hospital of Chongqing Medical University, Chongqing 400016, P.R. China; 2 Department of Geriatrics, Chengdu Fifth People’s Hospital, Chengdu, Sichuan 611130, P.R. China

**Keywords:** klotho, diabetes, renal hypertrophy, renal fibrosis, Rho-associated coiled-coil kinase

## Abstract

The present study aimed to investigate whether klotho gene delivery attenuated renal hypertrophy and fibrosis in streptozotocin-induced diabetic rats. A recombinant adeno-associated virus (rAAV) carrying mouse klotho full-length cDNA (rAAV.mKL), was constructed for *in vivo* investigation of klotho expression. Diabetes was induced in rats by a single tail vein injection of 60 mg/kg streptozotocin. Subsequently, the diabetic rats received an intravenous injection of rAAV.mKL, rAAV.green fluorescent protein (GFP) or phosphate-buffered saline (PBS). The Sprague-Dawley rat group received PBS and served as the control group. After 12 weeks, all the rats were sacrificed and ELISA, immunohistochemical and histological analyses, fluorescence microscopy, semi-quantitative reverse transcription-polymerase chain reaction and western blottin were performed. A single dose of rAAV.mKL was found to prevent the progression of renal hypertrophy and fibrosis for at least 12 weeks (duration of study). Klotho expression was suppressed in the diabetic rats, but was increased by rAAV.mKL delivery. rAAV.mKL significantly suppressed diabetes-induced renal hypertrophy and histopathological changes, reduced renal collagen fiber generation and decreased kidney hypertrophy index. In addition, rAAV.mKL decreased the protein expression levels of fibronectin and vimentin, while it downregulated the mRNA expression and activity of Rho-associated coiled-coil kinase (ROCK)I in the kidneys of the diabetic rats. These results indicated that klotho gene delivery ameliorated renal hypertrophy and fibrosis in diabetic rats, possibly by suppressing the ROCK signaling pathway. This may offer a novel approach for the long-term control and renoprotection of diabetes.

## Introduction

The klotho gene has been identified as an anti-aging factor (1). Several phenotypes of klotho mutant mice have been found to exhibit multiple disorders resembling human aging syndromes, including shortened life span, growth retardation, arteriosclerosis, skin and muscle atrophy and osteoporosis (2). In addition, overexpression of klotho extends the lifespan of mice by 20–30% (3).

Klotho is predominantly expressed in the choroid plexus of the brain and distal convoluted tubules of the kidney in normal individuals. A significant decline in the gene and protein expression levels of klotho have been reported in the kidneys of diabetic rats (4,5). Klotho overexpression has been found to modulate compensatory renal hypertrophy following nephrectomy by suppressing the insulin-like growth factor 1 (IGF-1) signaling pathway. This suppresses glomerulonephritis-induced renal fibrosis and preserves renal function (6,7). *In vitro* and *in vivo* experiments have demonstrated that reduced renal expression of klotho enhanced the activity of transforming growth factor-β1 (TGF-β1) and aggravated renal fibrosis by unilateral ureteral obstruction in mice (8). By contrast, overexpression of klotho has been shown to inhibit TGF-β1 signaling and suppress renal fibrosis (9). In addition, a previous study revealed that klotho suppressed renal fibrosis by inhibiting Wnt signaling in a model of kidney obstruction (10). Despite these observations, the role of klotho in diabetes-induced renal hypertrophy and fibrosis and the precise molecular mechanism remain to be elucidated.

The RhoA/Rho-associated coiled-coil kinase (ROCK) signaling pathway has been implicated in several diseases, including diabetic nephropathy. In diabetic milieu, high levels of glucose, advanced glycation endproducts, reactive oxygen species, the hexosamine pathway and oxidized low-density lipoprotein can activate the ROCK pathway in vascular and renal cells (11–17). In addition, the Rho/ROCK signaling pathway can be activated to mediate the effects of hormones, cytokines and mechanical stress involved in diabetic renal pathophysiology, including angiotensin II, aldosterone, vascular endothelial growth factor, TGF-β1 and mechanical stress (18). The increases in mesangial activity of RhoA and ROCK induced by high glucose levels are associated with an increased production and expression of collagen IV and the reorganization of fibronectin, vascular endothelial growth factor and actin (19,20). These effects are inhibited by the Y27632 or fasudil ROCK inhibitors or by the transfection of mesangial cells with an inactive RhoA mutant or RhoA-targeting small interfering RNA (19,20). Furthermore, in a study by Peng *et al*, ROCKI was demonstrated to prevent the high glucose-induced activation of activator protein 1, which is a transcription factor known to be involved in the upregulation of TGF-β1 and fibronectin genes (20). Inhibition of ROCK activity, through the administration of 30 mg/kg fasudil for 18 weeks, was found to attenuate the development of proteinuria, glomerulosclerosis and tubulointerstitial fibrosis and prevent the decrease in glomerular filtration rate in uninephrectomized streptozotocin-induced diabetic rats (21). In addition, this ROCK activity inhibition ameliorated the increased renal expression levels of TGF-β1, connective tissue growth factor and extracellular matrix (ECM), which were induced by diabetes (21). In non-diabetic models of kidney disease, the epithelial-mesenchymal transition (EMT), which is a process closely associated with the development of renal fibrosis, was found to be mediated by the Rho/ROCK signaling pathway (22,23). Furthermore, increased expression levels of EMT markers, including fibroblast specific protein 1, α-smooth muscle actin and vimentin (VIM), have been observed in uninephrectomized diabetic rats. ETM marker expression was attenuated by fasudil treatment, resulting in beneficial effects on renal fibrosis and reduced expression levels of TGF-β1 and connective tissue growth factor (21). In addition, Kikuchi *et al* (13) reported the beneficial effects of a high dose of fasudil (100 mg/kg) on proteinuria, glomerulosclerosis and interstitial fibrosis in a rat model of type 2 diabetes (Otsuka Long-Evans Tokushima Fatty rats). The aim of the present study was to examine whether klotho regulates diabetic-induced renal hypertrophy and fibrosis by suppressing the RhoA/ROCK pathway.

## Materials and methods

### Construction of a recombinant adeno-associated virus (AAV) vector containing the mouse klotho gene

A recombinant AAV vector carrying mouse klotho (mKL) full-length cDNA (rAAV.mKL) was prepared. Fresh mouse kidneys were obtained from the Experimental Animal Center of Chongqing Medical University (Chongqing, China). Kidneys were obtained within 5 min following sacrifice of a Kunming mouse (aged 5 weeks) through cervical dislocation following anesthetic with 10% chloral hydrate (HeChang Chemical Co., Ltd, Wuhan, China). The kidney tissue was then frozen in liquid nitrogen (Chongqing Jiangbei Manulife Gas Co., Ltd, Chongqing, China) and stored at −80°C overnight. The coding sequence of the mouse klotho mRNA was obtained from the GenBank database (NM_013823.2) and specific primers were designed (forward, 5′-CCGGAATTCATGCTAGCCCG-3′, and reverse, 5′-GCCGCTCGAGTTACTTATAACTTCTC-3′) to amplify the mouse klotho coding sequence. Following reverse transcription-polymerase chain reaction (RT-PCR), the PCR product was inserted into a pMD19-T vector (Takara Biotechnology Co., Ltd.) and verified by DNA sequencing using the Applied Biosystems 3730 DNA Sequencing system (Applied Biosystems Life Technologies, Foster City, CA, USA). Subsequently, the mouse klotho coding sequence was cut from the pMD19-T.mKL vector using *Eco*RI and *Xho*I and was subcloned into a pAAV-internal ribosome entry site (IRES)-humanized *Renilla* (Hr) green fluorescent protein (GFP) backbone between the *Eco*RI and *Xho*I sites. Following sequence verification, mKL expression was driven by a human cytomegalovirus intermediate-early promoter, followed by the IRESs, HrGFP and human growth hormone poly(A). The expression cassette was flanked by rAAV inverted terminal repeats. Large-scale vector production and purification was performed by the Vector Gene Technology Company, Ltd. (Shanghai, China), using rAAV.GFP as the control vector, prior to use in the present study. The vector batch titer was 3×10^8^ vector genomes (vg)/ml for AAV.mKL and 1×10^8^ vg/ml for rAAV.GFP.

### Animals

A total of 28 Male Sprague-Dawley (SD) rats (weighing between 200 and 250 g; aged 7 weeks) were purchased from the Experimental Animal Center of Chongqing Medical University (Chongqing, China) and housed under identical conditions with free access to food and water throughout the experiment. All the animal handling procedures were in accordance with the Regulations for The Administration of Affairs Concerning Experimental Animals. The study was approved by the Ethics Committee of the First Affiliated Hospital of Chongqing Medical University. All the surgical procedures were performed under anesthesia (10% chloral hydrate; 300 mg/kg; HeChang Chemical Co., Ltd.) and all efforts were made to minimize animal suffering. Diabetes was induced in the rats by a administering 60 mg/kg streptozotocin (Sigma-Aldrich, St. Louis, MO, USA) in 0.1 mol/l citrate buffer (pH 4.5) via a single tail vein injection (24,25). Age-matched rats were administered with an equivalent volume of citrate buffer via the same procedure and were used as the control group (SD rats). Streptozotocin-treated rats with blood glucose levels >16.7 mmol/l at one week after injection were considered diabetic (24).

Different treatment regimens were used in the present study, as follows: The diabetic rats, referred to as DM rats, were randomly divided into three groups (n=7 in each group). Prior to gene delivery, the body weight and fasting plasma glucose of the rats were measured, blood was obtained from the tail vein and serum was separated by centrifugation at 2,500 × g for 15 min for further analysis. The three groups of DM rats received a tail vein intravenous injection of rAAV.mKL (4×10^8^ particles/rat; 0.5 ml), rAAV.GFP (4×10^8^ particles/rat; 0.5 ml) or phosphate-buffered saline (PBS; 0.5 ml). The SD group (n=7) received PBS (0.5 ml) and served as the control. Body weights and fasting plasma glucose were measured every two weeks following gene delivery. At 12 weeks post-rAAV delivery, all the rats were sacrificed by exsanguination under anesthesia using 10% chloral hydrate (300 mg/kg). Fasting blood samples were obtained from the heart of the rats and the serum was separated by centrifugation at 2,500 × g for 15 min for further analysis. The kidneys were dissected and analyzed, as described in a subsequent section.

### ELISA

The plasma level of klotho was determined using an ELISA kit (R&D Systems, Minneapolis, MN, USA), according to the manufacturer’s instructions. In addition, the level of fasting plasma glucose was measured using a LifeScan One Touch UltraEasy Blood Glucose meter (LifeScan, High Wycombe, UK), according to the manufacturer’s instructions.

### Tissue preparation

The kidneys were washed with saline and weighed. The kidney weight and body weight ratio (g/g) ×10^3^ were calculated for each rat in order to determine the kidney hypertrophy index. A coronal section through the kidney midline at the level of the renal pelvis was fixed in 4% paraformaldehyde at 4°C for 24 h. Paraffin-embedded sections (4 *μ*m) were prepared for staining, using the Periodic acid-Schiff (PAS) staining and Masson’s trichrome staining kits, which were purchased from BoPei Biological Technology Co., Ltd (Chongqing, China) and immunohistochemical analysis, using Immunohistochemical (SP-9001) and Diaminobenzidine kits obtained from Zhongshan Golden Bridge Biotechnology Co., Ltd (Beijing, China). The remainder kidney sample was stored at −80°C for further analysis.

### Fluorescence microscopy

Unfixed kidney samples were frozen at −80°C and cut into 20-*μ*m sections. The expression of GFP in the kidneys was visualized with a fluorescein isothiocyanate filter using a Leica fluorescence microscope (DMIL4000, Leica Microsystems, Inc., Buffalo Grove, IL, USA) to determine the localization of the transgene.

### Immunohistochemical analysis

For immunohistochemical analysis, paraffin-embedded tissue sections (4 *μ*m) were incubated with peroxidase-blocking solution (Beijing Zhongshan Golden Bridge Biotechnology Co., Ltd) for 10 min, followed by incubation with protein blocker (Beijing Zhongshan Golden Bridge Biotechnology Co., Ltd) for 15 min. The sections were then incubated overnight at 4°C with the following primary antibodies: Polyclonal rabbit anti-rat/mouse klotho antibody(1:150; Abcam, Cambridge, MA, USA), polyclonal rabbit anti-rat fibronectin (FN) antibody (1:200; Bioworld Technology Inc., St Louis Park, MN, USA) and polyclonal rabbit anti-rat VIM antibody (1:100; Proteintech Group, Inc., Chicago, IL, USA). Next, the samples were incubated with a goat anti-rabbit immunoglobulin (Ig)G secondary antibody (1:6,000; Zhongshan Golden Bridge Biotechnology Co, Ltd) for 30 min at 37°C.

### Histological examination

For histological examination, the PAS-stained kidney tissue sections were analyzed under a microscope at x400 and the glomerular volumes were evaluated using an automatic inverted microscope (DMI4000, Leica Microsystems, Inc.). Glomerular tuft volumes were calculated from the midsection areas using the maximal planar area method (24). The glomerular volumes and total glomerular cell numbers of ≥25 randomly selected glomeruli from the renal cortex of each animal were measured (in total, 350–450 glomeruli from 5–7 rats per group). Masson’s trichrome staining was used to detect the accumulation of interstitial collagen fiber as a marker of renal fibrosis. The percentage of interstitial fibrosis of the total kidney area in a particular visual field or the collagen volume fraction was quantified. The samples were visualized and images were captured using a DMIL4000 Leica microscope. The images were then analyzed using the Image-Pro Plus 6.0 image analysis software (Media Cybernetics, Inc., Rockville, MD, USA) and the integrated optical density values of the metachromatic granules were recorded.

### Semi-quantitative RT-PCR

The expression levels of mKL, ROCKI and β-actin in the kidney tissues were examined using semi-quantitative RT-PCR. Total RNA was extracted from the freshly-frozen kidney samples using an RNAiso Plus kit (Takara Biotechnology Co., Ltd.) and cDNA was synthesized using a PrimeScript^®^ RT reagent kit (Takara Biotechnology Co., Ltd.), according to the manufacturer’s instructions. Subsequently, mKL, ROCKI and β-actin were amplified using a Multiplex PCR kit (Takara Biotechnology Co., Ltd.). The PCR (25 *μ*l) conditions were as follows: initial denaturation at 94°C for 5 min; 35 cycles of denaturation at 94°C for 30 sec, annealing at 60°C for 30 sec and elongation at 72°C for 35 sec; and final extension at 72°C for 5 min. The specific primers were designed based on published rat sequences from the GenBank database (Table I).

### Western blotting

The kidney tissues were homogenized in radioimmunoprecipitation assay lysis buffer (Beyotime Institute of Biotechnology, Jiangsu, China) containing 100 mg/ml phenylmethylsulfonyl fluoride and 1 mg/ml aprotinin and the lysate was centrifuged at 12,000 × g for 5 min. The protein concentration of the supernatant was quantified using a Bicinchoninic acid Protein Assay kit (Beyotime Institute of Biotechnology). Equal quantities of protein from each sample were separated by 6% sodium dodecyl sulfate-polyacrylamide gel electrophoresis and then electrotransferred onto polyvinylidene difluoride membranes (Beyotime Institute of Biotechnology). After blocking with 5% bovine serum albumin (Beyotime Institute of Biotechnology), the membranes were incubated with polyclonal rabbit anti-rat phosphorylated myosin phosphatase target subunit 1 (p-MYPT1; Thr696/Thr853) antibody (1:800; Cell Signaling Technology, Inc., Danvers, MA, USA), followed by incubation with horseradish peroxidase-conjugated affinipure goat anti-rabbit IgG secondary antibody (1:6,000; Zhongshan Golden Bridge Biotechnology Co., Ltd). Subsequently, the protein samples were visualized by enhanced chemiluminescence (Beyotime Institute of Biotechnology), exposed to an X-ray film (Kodak, Rochester, NY, USA) and developed with an X-ray processor (Chremi DOC XRS Volber Lourmat; Bio-Rad, Laboratories, Inc., Hercules, CA, USA). The protein band intensities were quantified using the Quantity One analysis software 4.6.2 (Bio-Rad Laboratories, Inc.) and the protein expression was normalized against the expression of β-actin.

### Statistical analyses

The data are presented as the mean ± standard error of the mean. Differences among the groups were assessed by one-way analysis of variance, followed by Tukey’s post-hoc multiple comparison test, using the SPSS software package for Windows (Version 17.0; SPSS, Inc., Chicago, IL, USA). P<0.05 was considered to indicate a statistically significant difference.

## Results

### Klotho gene delivery does not alter the blood glucose levels or body weight of DM rats

To investigate the effect of klotho gene delivery on DM rats, blood glucose levels were measured. The three DM rat groups received intravenous injection of rAAV.mKL, rAAV.GFP or PBS, wheareas the SD group received PBS (control group). The SD rats exhibited stable blood glucose levels (3.3–5.6 mmol/l) throughout the experimental period. However, the fasting blood glucose levels of the DM rats (18.10–25.70 mmol/l) were significantly higher compared with the SD rats, and this persistent hyperglycemia was accompanied by weight loss. In addition, the DM rats exhibited symptoms generally associated with diabetes, including dull fur, hair loss, lack of grooming, inactivity, polydipsia, polyuria and polyphagia. The results of the present study revealed that klotho gene delivery did not significantly alter the levels of blood glucose or the body weight of the DM rats (Table II).

### Klotho gene delivery reduces renal hypertrophy in DM rats

**Figure 1:**
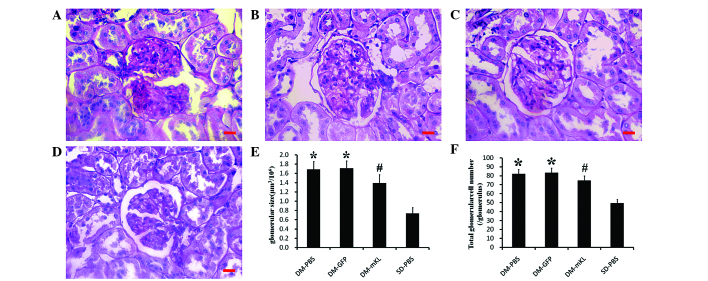
Representative microscopic images of the kidney tissues stained with periodic acid-Schiff (magnification, x400). The kidneys were obtained from DM rats treated with (A) PBS (DM-PBS group), (B) GFP (DM-GFP) and (C) mKL (DM-mKL). (D) SD rats were treated with PBS (SD-PBS; control). Scale bar=25 *μ*m. (E) Glomerular size and the (F) total glomerular cell number were determined in each group, by examining 350–450 glomeruli per group. The data are expressed as the mean ± standard error of the mean. ^*^P<0.05, vs. SD-PBS group; and ^#^P<0.05, vs. DM-PBS group. n=7 in each group. DM, diabetic; PBS, phosphate-buffered saline; GFP, green fluorescent protein; mKL, mouse klotho; SD, Sprague-Dawley.

**Figure 2:**
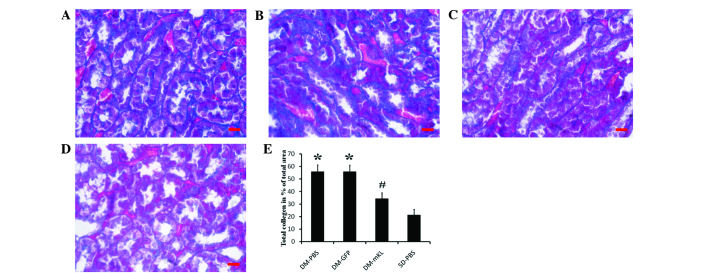
Representative microscopic images (magnification, x400) of kidney tissues stained with Masson’s trichrome, obtained from DM rats treated with (A) PBS (DM-PBS group), (B) GFP (DM-GFP group) and (C) mKL (DM-mKL group). (D) SD rats were treated with PBS (SD-PBS group). Scale bar=25 *μ*m. (E) Calculated collagen volume fraction. The data are expressed as the mean ± standard error of the mean. ^*^P<0.05, vs. SD-PBS group; and ^#^P<0.05, vs. DM-PBS group. n=7 in each group. DM, diabetic; PBS, phosphate-buffered saline; GFP, green fluorescent protein; mKL, mouse klotho; SD, Sprague-Dawley.

To understand how klotho gene delivery affects renal hypertrophy, histological examination of the kidney tissues was performed. The results demonstrated that the kidney hypertrophy index was significantly increased in the DM rats compared with the SD rats (P<0.05); however, the index was found to be markedly reduced by klotho gene delivery (DM-mKL; P<0.05; Table II). In addition, the rat kidney sections were stained with PAS for morphological analysis (Fig. 1A–D). An increase in the average glomerular volume and total glomerular cell number were observed in the DM rats compared with the SD rats (P<0.05), and this change was attenuated by klotho gene delivery (P<0.05; Fig. 1E and F). The kidney sections were also stained by Masson’s trichrome for tubulointerstitial collagen, which revealed that the degree of collagen deposition in the DM rats was more evident and fibrosis was enhanced compared with the SD rats. However, the degree of tubulointerstitial fibrosis in the rAAV.mKL-treated rats was markedly suppressed compared with the PBS-treated DM rats (Fig. 2A–D). Quantification of the tubulointerstitial collagen demonstrated that the collagen volume fraction was found to be significantly increased in the DM rats compared with the SD rats (P<0.05) and significantly decreased following klotho gene delivery (P<0.05; Fig. 2E). These data suggested that klotho gene delivery reduced renal hypertrophy in the DM rats.

### Klotho gene delivery increases the protein expression of klotho, but decreases the protein expression levels of FN and VIM in the kidneys of DM rats

**Figure 3:**
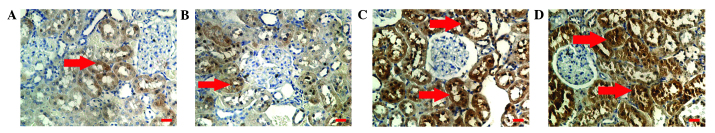
Immunohistochemical analysis images (magnification, x200) demonstrating the expression of klotho in the kidneys of DM rats treated with (A) PBS (DM-PBS group), (B) GFP (DM-GFP group) and (C) mKL (DM-mKL group), as well as (D) SD rats treated with PBS (SD-PBS group). Scale bar=25 *μ*m. The arrows indicate the protein expression of klotho in the renal tubule epithelial cells (brown staining). n=7 in each group. DM, diabetic; PBS, phosphate-buffered saline; GFP, green fluorescent protein; mKL, mouse klotho; SD, Sprague-Dawley.

**Figure 4:**
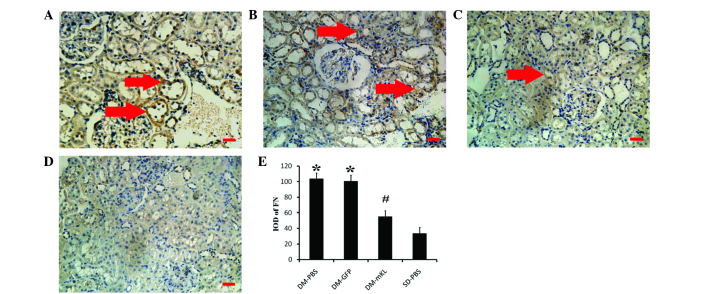
Immunohistochemical analysis (magnification, x200) of the expression of FN in kidney tissues obtained from DM rats treated with (A) PBS (DM-PBS group), (B) GFP (DM-GFP group) and (C) mKL (DM-mKL group), as well as (D) SD rats treated with PBS (SD-PBS group). Scale bar=25 *μ*m. The arrows indicate the protein expression of FN in the extracellular membrane of the renal tubule epithelial cells (brown staining). (E) Quantitative analysis of the protein expression of FN. The data are expressed as the mean ± standard error of the mean. ^*^P<0.05, vs. SD-PBS group; and ^#^P<0.05, vs. DM-PBS group. n=7 in each group. FN, fibronectin; DM, diabetic; PBS, phosphate-buffered saline; GFP, green fluorescent protein; mKL, mouse klotho; SD, Sprague-Dawley.

**Figure 5:**
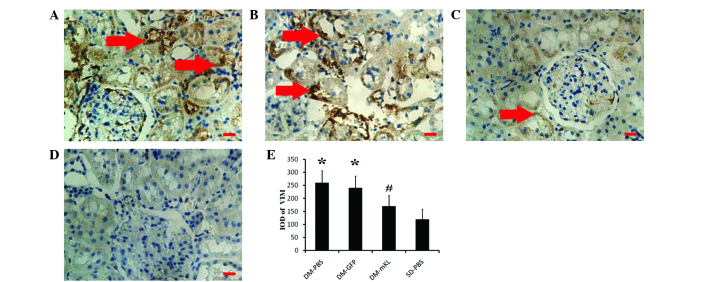
Immunohistochemical analysis (magnification, x200) of the expression of VIM in kidney tissues obtained from DM rats treated with (A) PBS (DM-PBS group), (B) GFP (DM-GFP group) and (C) mKL (DM-mKL group), as well as (D) SD rats treated with PBS (SD-PBS group). Scale bar=25 *μ*m. The arrows indicate the protein expression of VIM in the extracellular membrane of the renal tubule epithelial cells (brown staining). (E) Quantitative analysis of the protein expression of VIM. The data are expressed as the mean ± standard error of the mean. ^*^P<0.05, vs. SD-PBS group; and ^#^P<0.05, vs. DM-PBS group. n=7 for each group. VIM, vimentin; DM, diabetic; PBS, phosphate-buffered saline; GFP, green fluorescent protein; mKL, mouse klotho; SD, Sprague-Dawley.

In order to investigate the effect of klotho gene delivery on the protein expression levels of klotho, FN and VIM, immunohistochemical analysis was performed. The results revealed that the protein expression of klotho (brown staining) was localized in the renal tubule epithelial cells (Fig. 3A–D). In addition, the renal expression of klotho was lower in the DM rats compared with the SD rats. However, klotho gene delivery increased the protein expression of klotho (dark brown staining) in the renal tubule epithelial cells of the DM rats (Fig. 3C). In addition, immunohistochemical analysis indicated that the protein expression of FN (brown staining) was localized in the extracellular membrane of the renal tubule epithelial cells (Fig. 4A–D). The renal expression of FN was found to be higher in the DM rats compared with the SD rats. However, the expression of FN in the DM rats treated with rAAV.mKL was lower compared with the DM rats treated with PBS or rAAV.GFP (Fig. 4E). Similar to FN, the protein expression of VIM (brown staining) was also localized in the extracellular membrane of the renal tubule epithelial cells (Fig. 5A–D). The renal expression of VIM was found to be higher in the DM rats compared with the SD rats. However, the expression of VIM in the DM rats treated with rAAV.mKL was lower compared with the DM rats treated with PBS or rAAV.GFP (Fig. 5E). These results indicated that klotho gene delivery increased the protein expression of klotho, but decreased the protein expression levels of FN and VIM in the kidneys of DM rats.

### Klotho gene delivery suppresses ROCK activation in DM rats

**Figure 6:**
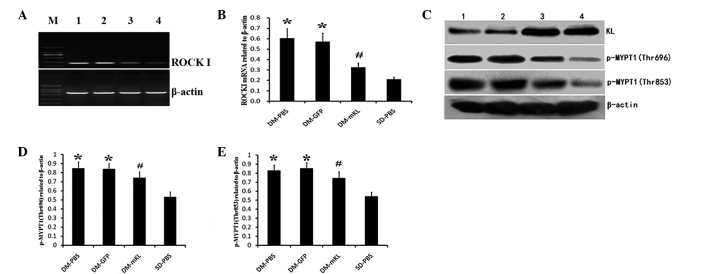
mRNA expression of ROCKI in kidney tissues (A) determined by semi-quantitative reverse transcription-polymerase chain reaction and (B) obtained by quantitative analysis. Protein expression levels of p-MYPT1, (C) determined by western blotting, for (D) p-MYPT1 (Thr696) and (E) p-MYPT1 (Thr853). The data are expressed as the mean ± standard error of the mean. ^*^P<0.05, vs. SD-PBS group; and ^#^P<0.05, vs. DM-PBS group. n=7 for each group. Groups: M, DL 2000 DNA Marker; 1, DM-PBS; 2, DM-GFP; 3, DM-mKL; and 4, SD-PBS. ROCKI, Rho-associated coiled-coil kinase 1; p-MYPT1, phosphorylated myosin phosphatase target subunit 1; DM, diabetic; PBS, phosphate-buffered saline; GFP, green fluorescent protein; mKL, mouse klotho; SD, Sprague-Dawley.

In order to investigate the activation of ROCK and the protein expression of p-MYPT1 (Thr696/Thr853), RT-qPCR and western blotting were performed. The mRNA expression level of ROCKI was analyzed in the kidney tissues of each experimental group and was found to be significantly higher in the DM rats compared with the SD rats (P<0.05). However, following treatment with rAAV.mKL, the mRNA expression of ROCKI was significantly suppressed compared with the PBS- or rAAV.GFP-treated DM rats (P<0.05; Fig. 6A and B). Consistent with the semi-quantitative RT-PCR results, western blotting revealed that p-MYPT1 (Thr696/853) was significantly elevated in the DM rats (Fig. 6C) and was suppressed following treatment with rAAV.mKL (P<0.05; Fig. 6D–E). These results indicated that klotho gene delivery suppressed the mRNA expression of ROCKI, and thus, the activation of the ROCK signaling pathway in the DM rats.

### Klotho gene delivery upregulates plasma levels of the klotho protein in DM rats

**Figure 7:**
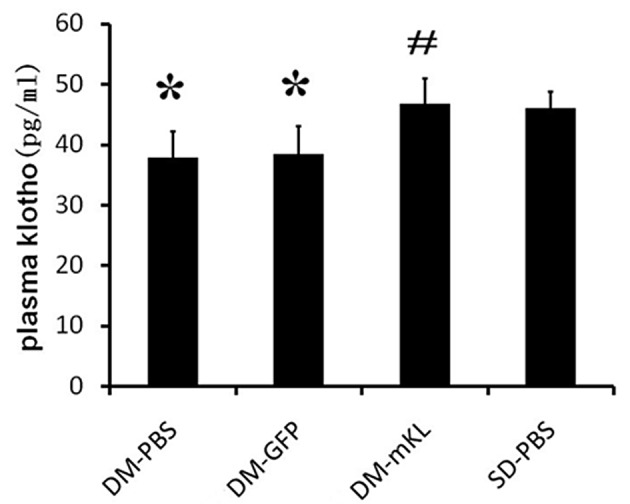
Quantitative analysis of the concentration of plasma klotho induced by klotho gene delivery in different groups. Values are expressed as the mean ± standard error of the mean. ^*^P<0.05, vs. SD-PBS group. ^#^P<0.05, vs. DM-PBS group. n=7 in each group. DM, diabetic rats; PBS, phosphate-buffered saline; GFP, green fluorescent protein; mKL, mouse klotho; SD, Sprague-Dawley rats.

In order to determine the plasma levels of klotho protein, ELISA was performed. The data indicated that the levels of plasma klotho were lower in the DM rats compared with the SD rats. Klotho gene delivery increased the plasma klotho levels in the rAAV.mKL-treated rats compared with the DM rats treated with PBS and rAAV.GFP (Fig. 7). Therefore, klotho gene delivery was found to upregulate the levels of klotho protein in the plasma of DM rats.

### rAAV results in long-term mRNA expression of GFP and mKL in the rat kidneys

**Figure 8:**
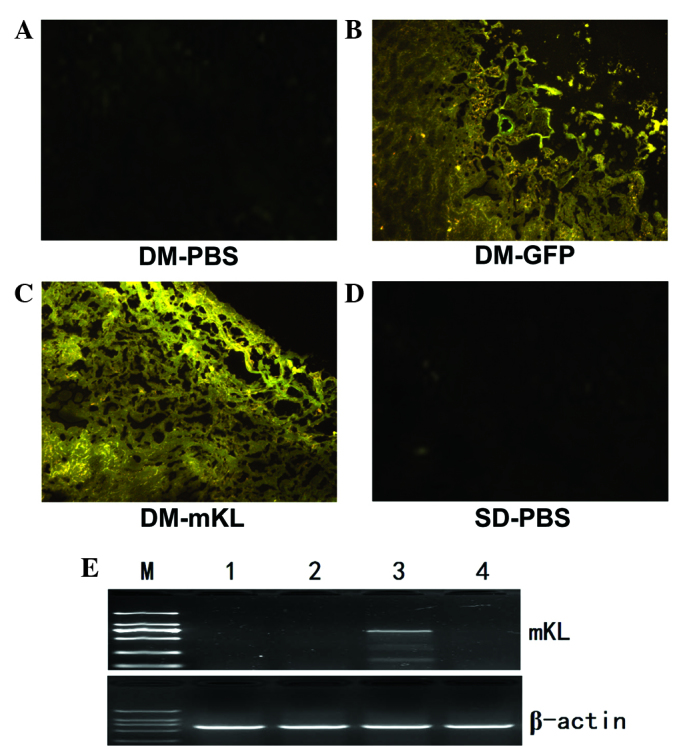
mRNA expression of GFP and mKL in kidney tissues. Fluorescent photomicrographs of GFP expression in the kidneys of DM rats treated with (A) PBS, (B) rAAV.GFP or (C) rAAV.mKL and (D) SD rats treated with PBS. (E) Semi-quantitative reverse transcription-polymerase chain reaction detection of the mRNA expression of mouse klotho in the kidneys of rats treated with rAAV.mKL using a mouse klotho-specific primer. Groups: M, DL 2000 DNA Marker; 1, DM-PBS; 2, DM-GFP; 3, DM-mKL; and 4, SD-PBS. DM, diabetic; PBS, phosphate-buffered saline; GFP, green fluorescent protein; mKL, mouse klotho; SD, Sprague-Dawley; AAV, adeno-associated virus.

To visualize the mRNA expression levels of GFP and mKL in the rat kidneys, fluorescence microscopy was used. In the DM-GFP and DM-mKL groups, GFP expression was detected (Fig. 8B and C), indicating that GFP was expressed in the rats 12 weeks after the delivery of rAAV.GFP and rAAV.mKL. By contrast, no GFP expression was detected in the kidneys of the DM-PBS or SD-PBS groups. In addition, mouse klotho was highly expressed in the kidney of the rAAV.mKL-treated rats 12 weeks after gene delivery (Fig. 8E), indicating the successful delivery of the mouse klotho gene. However, no mouse klotho mRNA was observed in any other group. These results demonstrated that rAAV resulted in long-term transgene expression.

## Discussion

The main characteristic pathological features of diabetic kidney damage are early glomerular hypertrophy, glomerular capillary basement membrane thickening and gradual accumulation of ECM in the mesangial and renal tubular interstitial area (26). The inherent structure of the kidney is then damaged and eventually results in glomerular sclerosis and interstitial fibrosis that leads to kidney failure. In the present study, DM rats were transfected with exogenous rAAV.mKL and the klotho gene was found to have no effect on body weight or fasting glucose level. However, the klotho gene was able to inhibit kidney hypertrophy and fibrosis, delay the pathological changes of diabetic kidney, including glomerular hypertrophy, glomerular capillary basement membrane thickening, mesangial and renal tubular interstitial area collagen fibrosis, and reduce the kidney hypertrophy index of the DM rats. Therefore, increased klotho gene expression was shown to have a protective effect on the kidney of DM rats.

Renal hypertrophy is an early characteristic pathological change occurring in diabetic nephropathy and is closely associated with late renal fibrosis (27). The mammalian target of rapamycin (mTOR) pathway is the most important downstream signaling pathway of compensatory renal hypertrophy following nephrectomy. Rapamycin is an mTOR inhibitor, which inhibits compensatory tubular cell hypertrophy. Furthermore, compensatory renal hypertrophy is suppressed in ribosomal protein S6 knock-out mice, which do not exhibit compensatory renal growth or tubular cell proliferation following nephrectomy (28,29). Therefore, mTOR is essential in mediating increased RNA and protein synthesis in compensatory renal hypertrophy (29). The increase of reactive oxygen species caused by NAD(P)H oxidase activation is important in renal glomerular hypertrophy signal transduction and cell proliferation following unilateral nephrectomy (30). Nagasu *et al* (6) investigated the compensatory renal hypertrophy induced by unilateral nephrectomy in a klotho transgenic mouse model. The results revealed that overexpression of the klotho gene restrained the activation of mTOR and the increase in reactive oxygen species resulting from NAD(P) H oxidase activation and, thus, reduced renal hypertrophy (6). These effects are achieved by the inhibition of the insulin-like growth factor-1 (IGF-1) signaling pathway by the klotho gene. However, whether the overexpression of the klotho gene inhibits diabetes-induced renal hypertrophy through inhibition of the IGF-1 signaling pathway remains to be elucidated.

Renal fibrosis, including glomerular sclerosis and renal interstitial fibrosis, is the final common pathway in the majority of cases of end-stage renal disease. FN, an adhesion molecule, is an important component of ECM and its expression can be increased with a prolonged duration of DM. In the glomerular mesangial and renal tubular interstitial areas, excessive ECM deposition results in glomerular sclerosis and renal interstitial fibrosis (31,32). The present study demonstrated that exogenous rAAV.mKL transfection inhibited the expression of FN and reduced the generation and accumulation of ECM in the kidneys of the DM rats.

EMT is a process by which fully differentiated epithelial cells undergo transition to a fibroblast phenotype (33). The migration of epithelial cells from the tubular structure into the interstitium, where matrix is produced (including collagen types I and IV and FN), contributes to the formation of renal fibrosis (34). Previous studies have demonstrated that, in addition to renal tubular epithelial cells, sertoli and endothelial cells also undergo phenotypic transformations, which are termed podocyte- and endothelial-mesenchymal transitions, respectively (35,36). VIM is an intermediate filament constituent of the cytoskeleton of mesenchymal cells. The expression of VIM is associated with the cell phenotype, including shape, motility and adhesion. VIM is an EMT-specific marker and its expression is closely associated with the degree of EMT (37). Previous studies have revealed that a reduced expression level of klotho in the kidneys aggravates renal interstitial fibrosis in mice induced by unilateral ureteral obstruction (8). In addition, the secreted klotho suppresses the TGF-β1-induced EMT response in cultured cells, including decreased expression levels of epithelial markers, increased expression levels of mesenchymal markers and/or increased cell migration. Furthermore, secreted klotho inhibits Wnt and IGF-1 signaling, which promotes EMT (9). The present study demonstrated that exogenous rAAV.mKL transfection in DM rats decreased the protein expression of VIM and reduced the pathological changes in the kidney and collagen fiber formation. Therefore, as discussed earlier, the klotho gene may reduce the progression of diabetes-induced renal fibrosis by inhibiting ECM and EMT.

Previous *in vivo* and *in vitro* experiments have demonstrated that the activation of ROCK is involved in the occurrence and development of diabetic nephropathy (18). Kolavennu *et al* and Peng *et al* identified that the expression of FN in mesangial cells cultured in a high glucose environment was increased upon activation of the ROCKI signaling pathway (19,20). In addition, Komers *et al* observed that activation of the ROCK signaling pathway in kidneys with diabetic nephropathy increased the expression of VIM and aggravated renal fibrosis; however, ROCK-specific inhibitors reduced the process of renal fibrosis (21). Furthermore, a previous study demonstrated that administering fasudil in rats exhibiting diabetic cardiomyopathy inhibited the mRNA transcription and protein activity of ROCKI and thus, reduced diabetic myocardial fibrosis, oxidative stress and the apoptosis of myocardial cells (38). The results of the present study demonstrated that increased expression of klotho gene in the kidneys of DM rats inhibited the protein expression of FN and VIM, which may be associated with the inhibition of the mRNA expression and protein activity of ROCKI.

A previous study revealed that RhoA/ROCK signaling pathway activation is closely associated with the expression of klotho (39). However, the mechanism underlying the regulation of the mRNA transcription and protein activity ROCKI by klotho remains unclear. The increased mRNA transcription and protein activity of ROCK have been associated with the remnant lipoprotein and inflammatory factors, including angiotensin II and interleukin-1, which stimulate the protein kinase C/nuclear factor (NF)-κB pathway (40,41). In addition, Zhao *et al* identified that increased exogenous klotho protein levels in db/db diabetic mice inhibited NF-κB activity (42). Therefore, the present study hypothesized that: i) Klotho may inhibit the mRNA transcription and protein activity of ROCKI, possibly by inhibiting NF-κB activity; ii) klotho may produce two proteins, including the membrane and secretion klotho proteins. Membrane klotho protein functions as an obligatory co-receptor for fibroblast growth factor 23, whereas the function of secretion klotho protein can vary with the circulation to affect various signal transduction pathways (43–45). However, further studies are required to elucidate whether the membrane and secretion klotho proteins have associated sites for direct binding with the small GTPase protein, RhoA, which indirectly affects the activity of ROCK.

Several studies have demonstrated that the gene and protein expression levels of klotho are serially declined in chronic renal failure patients and diabetic rats (4,5,46). The present study demonstrated that transfection with exogenous rAAV.mKL stabilized the expression of klotho for at least 12 weeks and upregulated the protein expression of klotho in the serum of DM rats. These results provide a basis for understanding the mechanism by which klotho functions in diabetes and associated diseases and may be helpful in the development of a novel long-term treatment for age-related diseases, including diabetes.

Klotho protein is unique since it inhibits four signaling pathways simultaneously, offering a major advantage over numerous individual inhibitors in clinical and preclinical development, including IGF-1 receptor antibodies, tyrosine kinase (47) and Wnt signaling inhibitors (48), TGF-β1 neutralizing antibodies, soluble TGF-βR2, TGF-β receptor kinase inhibitors (49,50) and ROCK signaling inhibitors. In addition, klotho therapy is considered to be safe since overexpression of klotho extends the lifespan of mice (3). Therefore, klotho protein may be a novel therapeutic agent for the treatment of tissue hypertrophy and fibrosis with a unique mechanism of action.

In conclusion, the present study demonstrated that exogenous rAAV.mKL transfection in DM rats upregulated the expression of klotho and delayed the progression of renal hypertrophy and fibrosis induced by diabetes. To a certain extent, these results may be due to klotho suppressing the ROCK signaling pathway. However, further studies are required to understand how klotho affects the mRNA transcription and protein activity of ROCKI.

## Figures and Tables

**Table I:** Primer sequences used in reverse transcription-quantitative polymerase chain reaction.

Primer target	Primer	Primer sequence	Product length (bp)
mKL	Sense	5′-GGGTCACTGGGTCAATCT-3′	710
	Antisense	5′-GCAAAGTAGCCACAAAGG-3′	
ROCKI	Sense	5′-ATCCACCAGGAAGGTTTATGC-3′	226
	Antisense	5′-AGGCACATCGTAGTTGCTCAT-3′	
β-actin	Sense	5′-ACTGTGCCCATCTACGAGG-3′	678
	Antisense	5′-GAAAGGGTGTAACGCAACTA-3′	

ROCKI, Rho-associated coiled-coil kinase I; mKL, mouse klotho.

**Table II:** Effect of klotho gene delivery on fasting plasma glucose, body weight and kidney weight.

Group	n	FPG (mmol/l)	BW (g)	KW (g)	KHI (g/g × 1,000)
DM-PBS	7	21.86±1.94^a^	495.58±39.66^a^	3.25±0.34^a^	6.56±0.33^a^
DM-GFP	7	20.88±1.94^a^	492.92±74.02^a^	3.21±0.48^a^	6.52±0.29^a^
DM-mKL	7	20.44±1.59^a^	497.57±64.69^a^	3.04±0.61^a^	6.06±0.52^b^
SD-PBS	7	4.63±0.42	586.50±64.18	2.03±0.21	3.47±0.19

Data are expressed as the mean ± standard error of the mean.

a P<0.05, vs. SD-PBS group; and

b P<0.05, vs. DM-PBS group. FPG, fasting plasma glucose; BW, body weight; KW, kidney weight; KHI, kidney hypertrophy index; DM, diabetic rats; PBS, phosphate-buffered saline; GFP, green fluorescent protein; mKL, mouse klotho; SD, Sprague-Dawley rats.
